# “We built the ship as we sailed it”: exploring public health outreach targeting communities shaped by migration in Stockholm, Sweden during the COVID-19 pandemic

**DOI:** 10.1186/s12913-026-15396-z

**Published:** 2026-08-18

**Authors:** Mattias Strand, Baidar Al-Ammari, Önver Cetrez, Soorej Jose Puthoopparambil, Sofie Bäärnhielm

**Affiliations:** 1 Centre for Psychiatry Research, Department of Clinical Neuroscience, Karolinska Institutet, Stockholm, Sweden; 2 Transcultural Centre, Stockholm Health Care Services, Region Stockholm, Solnavägen 1E, Stockholm, 113 65 Sweden; 3 Department of Theology, Uppsala University, Uppsala, Sweden; 4 Global Health and Migration Unit, Department of Women’s and Children’s Health, Uppsala University, Uppsala, Sweden

**Keywords:** COVID-19, Health communication, Public health, Community involvement, Migration, Migrant health, Superdiversity

## Abstract

**Background:**

Effective health crisis communication requires culturally sensitive, community-engaged approaches, particularly for reaching vulnerable populations. The COVID-19 pandemic exposed significant gaps in public health preparedness for communities shaped by migration. This study explores how public health staff in Stockholm, Sweden designed and delivered crisis communication targeting migrant populations during the pandemic.

**Methods:**

A qualitative study was conducted using eight individual in-depth interviews and three focus group discussions with 21 participants involved in planning, producing, and disseminating COVID-19 public health messages in Stockholm. Participants included public health strategists, communications officers, and frontline healthcare staff. Interview data were analyzed using thematic analysis.

**Results:**

Four main themes emerged from the data, revealing a temporal evolution in outreach strategies. Initially, efforts focused heavily on language translation of standardized health messages. However, participants recognized this approach as insufficient and gradually shifted toward more dialogue-based engagement. Key developments included: (a) establishing trust through active involvement of community stakeholders such as religious leaders, neighborhood organizers, and social media influencers; (b) addressing structural barriers including overcrowded housing, precarious employment, and digital exclusion rather than assuming non-adherence reflected poor understanding; (c) counteracting stigma related to infection and vaccination; and (d) fostering creative problem-solving despite organizational rigidity and unclear coordination. Participants emphasized the importance of listening to the concerns in the community and co-developing solutions rather than simply disseminating information. However, many expressed concern that networks established during the pandemic and insights gained about structural inequalities were already being lost as systems “returned to normal”.

**Conclusion:**

Public health authorities must move beyond one-way messaging toward sustained community partnership and dialogue-based engagement. Effective crisis preparedness requires addressing social determinants of health and building community networks during non-crisis periods, rather than relying on ad-hoc solutions during emergencies. Without institutional commitment to translating crisis experiences into lasting structural changes, authorities risk repeating past mistakes and eroding public trust—a critical concern given the likelihood of future health crises.

**Trial registration:**

The study protocol was preregistered on the Open Science Framework (osf.io/rt47j) on March 22, 2022.

**Supplementary Information:**

The online version contains supplementary material available at 10.1186/s12913-026-15396-z.

## Background

Public health crisis communication refers to the process by which public health authorities and organizations communicate with the public during an actual or potential health threat, with the aim of reducing uncertainty and enabling informed, protective decision-making. Existing frameworks for such communication highlight its phased, situation-specific, and culturally sensitive nature, particularly regarding effective community outreach and engagement with vulnerable populations [1–4]. A critical component in public health outreach is the necessity of establishing trust, defined as confidence in the competence, fairness, transparency, and accountability of leadership, among individuals and communities in times of crisis. Importantly, this capacity can be strengthened by the use of communication strategies that engage in active dialogue with vulnerable populations and acknowledge their priorities and lived realities [4].

The coronavirus disease 2019 (COVID-19) pandemic presented enormous public health outreach and communication challenges worldwide. The impact of *crisis psychology*—i.e., the notion that people tend to process and act on information differently under stress than they would in a non-crisis context—is well-recognized in the field [2]. As people have easy access to information through social media and global media outlets, locally relevant official public health recommendations for COVID-19 containment competed with outright misinformation and information not relevant to the local context in gaining public attention [5, 6]. Reaching and establishing trust among minoritized communities, such as culturally and linguistically diverse populations with a migration background, proved to be a complex task in many settings [7–9]. These challenges should not be exaggerated, however; when queried, individuals with a migration background often reported receiving adequate information and mostly adhering to local recommendations for pandemic containment [10, 11]. Nonetheless, migrant and ethnically minoritized populations have consistently displayed higher levels of COVID-19-related morbidity and mortality, which may be more closely associated with social determinants of health such as poverty, structural racism, and asymmetric access to healthcare [12–14] than with being uninformed.

In Sweden, individuals with a migration background (i.e., people who have migrated or whose parents have migrated from another country) in socioeconomically underprivileged neighborhoods were clearly overrepresented among the infected and deceased during the COVID-19 pandemic [15]. For the duration of the pandemic, a substantially higher risk of COVID-19-related intensive care unit admission was seen among migrants from Africa, Middle-East, Asia, South America, and other European countries compared to the Swedish-born population [16]. Furthermore, Swedish COVID-19 vaccination coverage was substantially lower in the same socioeconomically underprivileged neighborhoods that had been hardest hit during the first wave of the pandemic [17]. Clearly, health crisis preparedness in terms of community outreach was not sufficient. For example, despite a strong Swedish public health policy emphasis on social determinants of health, the pre-COVID-19 epidemic preparedness plan for the Stockholm region did not include explicit measures for reaching populations with a migration background and other vulnerable groups [18].

The Swedish public health system is usually considered highly decentralized, with a high degree of autonomy granted to 21 regional authorities [19]; this study focuses on public health outreach in the Stockholm region during the COVID-19 pandemic. Stockholm is the largest city in Sweden, with a population of 2.5 million of which 37% are either born abroad or have two parents born abroad [20]. Significant numbers of non-European migrants in the region originate from countries such as Iraq, Iran, Syria, Afghanistan, Eritrea, Somalia, India, Pakistan, China, and Thailand. The aim of this study was to explore the experiences of staff involved in designing, organizing, and delivering public health crisis communication in Stockholm, Sweden during the COVID-19 pandemic, with a focus on outreach efforts targeting populations with a migration background. Ultimately, this can contribute to strengthening the capacity for effective outreach in the event of future health crises.

## Methods

### Study design and participants

For this study, a qualitative research design was employed, based on eight individual in-depth interviews and two focus group interviews with staff involved in designing, organizing, and delivering public health crisis communication in Stockholm, Sweden during the COVID-19 pandemic. The Transcultural Center (www.transkulturelltcentrum.se) is a resource center for migrant health in Region Stockholm, the public body responsible for healthcare within Stockholm county. Based on insights from the Transcultural Center’s participation in regional COVID-19 pandemic response networks, we reached out to administrative staff at different levels in the region who had held some responsibility for planning, producing, and/or disseminating public health messages directed toward migrants and other groups with another first language than Swedish during the pandemic and requested to interview them about their experiences. This included public health professionals involved in strategic outreach work, communications and media officers, and frontline staff responsible for putting official communication guidelines into practice in primary care. Participants could also suggest additional people to interview; several of them did, although the names mentioned were all already on our list of potential participants, underscoring the limited number of eligible participants involved in public health crisis communication in Region Stockholm during the COVID-19 pandemic. A total of 22 individuals were invited to participate, and all but one agreed. Given the small number of individuals involved in the public outreach activities, we are only able to provide participant characteristics in aggregated form to avoid any risk of identification. Of the 22 participants, six can be characterized as senior-level strategists (employed by Region Stockholm or engaged on a consultancy basis), while the remaining 16 worked more directly on translating strategy into action, either as mid-level professionals or frontline staff. Two participants were employed by the Transcultural Center at the time of the COVID-19 pandemic.

The combination of data-collection through individual interviews and focus group discussions was chosen primarily for practical reasons rather than to leverage the distinct methodological strengths typically associated with each approach. Specifically, focus groups were conducted with participants who worked within the same office and had limited availability for individual scheduling; group sessions therefore offered a more time-efficient way to collect data from these participants than arranging separate interviews with each. The same interview guide was used across both individual interviews and focus groups, with minor adaptations to accommodate group format where necessary.

### Procedures

An interview guide was developed for this study, focusing on participant experiences of strategic outreach, communication strategies, and collaboration with various stakeholders during the COVID-19 pandemic. The interview guide was formulated so as to assure participants that their individual performances were not being evaluated and that the aim was to identify overarching themes that might guide similar health crisis response strategies in the future. A translated version of the interview guide is provided as supplementary material. Individual in-depth interviews were conducted with eight participants; furthermore, 13 participants (all frontline workers) were interviewed through three focus group sessions. SB conducted four individual and two focus groups interviews, MS conducted four individual interviews, and ÖC and BA jointly conducted one focus group interview. The interviews and focus groups lasted between 35 and 70 min. All interviews were conducted in Swedish. The interviews were audio recorded and transcribed verbatim. The transcripts were pseudonymized by omitting or changing details that could risk identifying the individual participants.

The transcribed interview data were analyzed employing a codebook thematic analysis approach, combining the framework outlined by Braun and Clarke [21] with elements of conventional qualitative content analysis [22]. In a first step, all authors familiarized themselves with the interview data. Second, two team members (BA and MS) coded the data together, identifying meaning units and developing preliminary categories and themes. Here, an inductive, bottom-up principle was applied, avoiding as far as possible preconceived notions regarding topics that might prove meaningful in the participant narratives. This process resulted in a preliminary codebook, which was applied provisionally to the dataset and refined through repeated discussion between the two coders as new codes emerged or existing codes were merged, split, or redefined. Third, this preliminary analysis—including the codebook itself, which continued to evolve substantially as categories and themes were reorganized—was iteratively reviewed and reworked jointly by all team members, using mapping techniques in order to achieve an optimal structure for describing the data. Finally, the findings were presented and contextualized, and illustrative quotes were chosen, avoiding details that could risk identification of participants.

### Ethics and preregistration

All procedures were conducted in accordance with the Helsinki declaration. The study was approved by the Swedish Ethical Review Authority (No. 2022-01637-01). Written and verbal informed consent was obtained from all participants. Given the sensitive nature of the data involved in this study—including information on ethnicity and health—and the potential risks to participants such as breaches of integrity and stigmatization, we conducted data collection and preservation with the utmost care. All procedures strictly followed ethical guidelines and university-specific regulations concerning data management. Study design and reporting were guided by the Standards for Reporting Qualitative Research framework [23] to ensure rigor and transparency. The study protocol has been preregistered on the Open Science Framework (osf.io/rt47j).

## Results

**Fig. 1:**
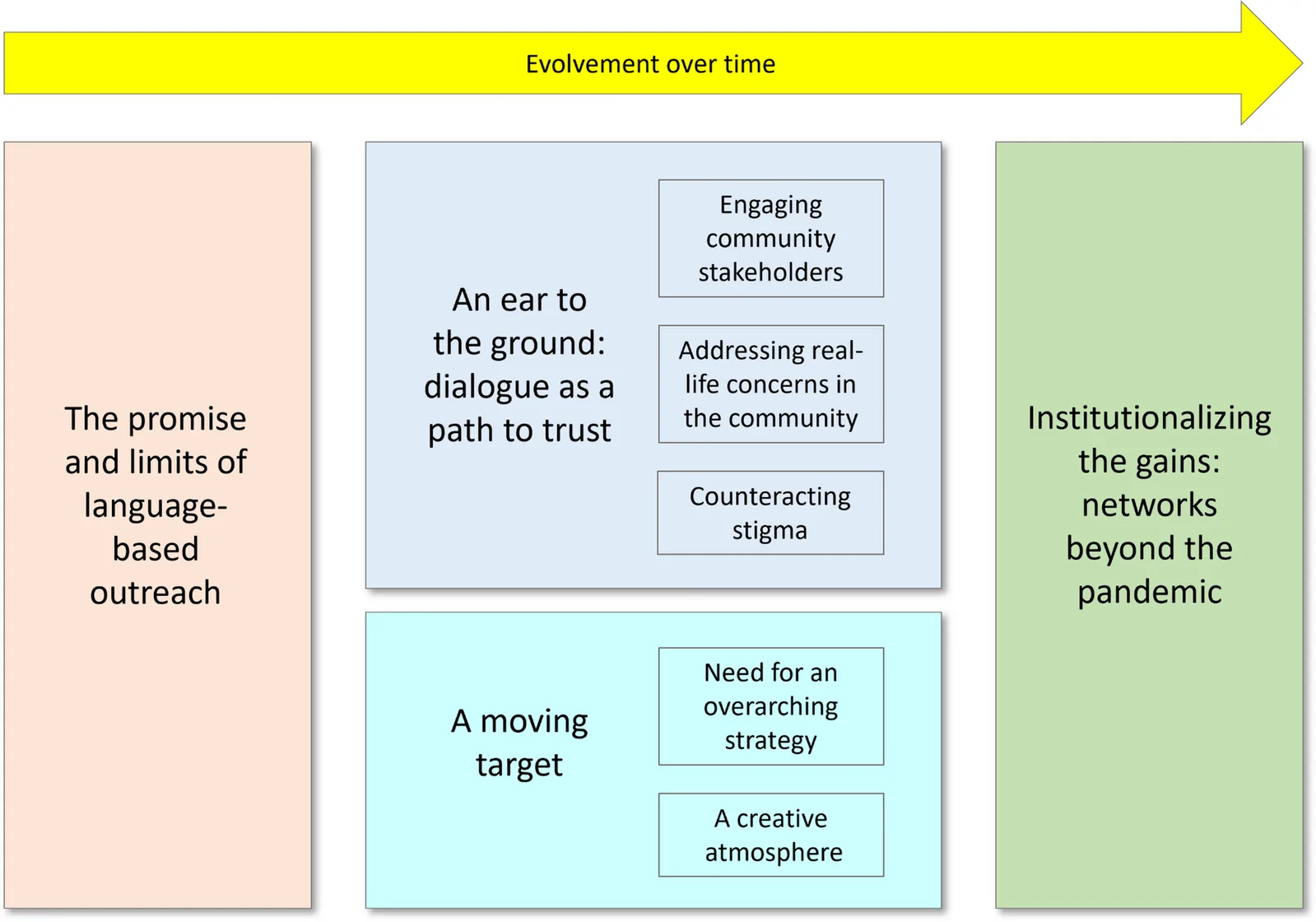
Themes and subthemes

Four overarching themes were identified in the interview data. As depicted in Fig. 1, the participant narratives also involved a notion of temporal evolvement, so that novel themes arose and became more important as the pandemic developed over time, allowing for iterative learning. This temporal pattern emerged inductively from participants’ own retrospective descriptions of how their work evolved over the course of the pandemic, rather than from a systematic analysis of time markers or a structured comparison across interviews conducted at different time points. Two of the four themes are further organized into subthemes, presented as subheadings below, which capture more specific aspects of the broader theme; the remaining two themes are reported without further subdivision.

### The promise and limits of language-based outreach

As it quickly became clear that communities with many residents with a migration background in the Greater Stockholm area displayed higher levels of COVID-19-related morbidity, there was a large emphasis on ensuring that the official public health recommendations for pandemic containment were made available in other languages than Swedish. Some of these translation efforts were initiated by higher-level health authorities in the region; this included, for example, texting information videos with chief medical officers in Arabic (the second most common first language in Sweden) and translating widely distributed posters with public health advice into languages other than Swedish that are spoken in Stockholm. At other times, the task of translating information material fell on frontline staff in primary care, who had to improvise relying on their own multilingualism. The participants generally agreed about the usefulness of translating public health recommendations into major languages spoken in populations with a migration background, such as Arabic, Somali, Tigrinya, Dari/Farsi, and English. The emotionally potentially charged content underscored this need; for health and safety decision-making, participants underscored the need to provide alternative options for those whose Swedish or English skills, while adequate for daily communication, are insufficient for complex discussions. Furthermore, they stressed that providing translations of official public health recommendations can prevent the spread of misinformation or information that is not contextually relevant:

When government agencies claim that some people rely on media reports from their countries of origin, I’m thinking that it’s not such a huge surprise because your website isn’t available in their language. And the media briefings aren’t translated. How can we blame someone for not looking for information where there is none? [from interview 5]

However, some also voiced concern about an overemphasis on translation—especially translations targeting groups who are typically bilingual in Swedish—at the expense of other, potentially more urgent activities:

Is it really helpful to translate into [smaller minority language, whose speakers are mostly also fluent in Swedish] and god knows what? […] I think we translated into more than 30 languages, and it was probably rather redundant. Translations don’t solve everything, but it might look like it does from the outside, if you know what I mean. [from interview 4]

In sum, the initial focus on language and translation was largely seen as necessary and useful, but participants thought that it might also have overshadowed other potentially helpful interventions. These aspects, more closely associated with structural barriers to pandemic containment, are addressed in the next theme.

### An ear to the ground: dialogue as a path to trust

As the COVID-19 pandemic continued, the major focus on language and translation was seen as insufficient. Over time, it became obvious that reported difficulties in communities with many residents with a migration background in fully adhering to official public health recommendations did not primarily stem from poor understanding in terms of language, but from structural barriers such as overcrowded housing and not having the option of working from home. This novel insight, in turn, necessitated a shift away from one-way communication of translated public health information toward community dialogue and involvement, allowing for an increased attention to the real-life experiences of community members. Here, three subthemes were identified: the importance of trust-building, addressing major actual concerns in the community, and counteracting stigma.

#### Engaging community stakeholders

The participants described how simply providing factual advice on pandemic-related issues did not suffice; in order to reach populations with a migration background in particular, health authorities need to find ways to build and maintain trust among the public. This might be even more important among migrants from authoritarian countries who may not share an intuitive understanding of government agencies as well-intentioned. A crucial aspect of successful outreach was the active involvement of community stakeholders. This typically included neighborhood organizers, religious leaders, and representatives of migrant organizations, but also more unconventional actors such as social media influencers:

I remember filming a video with this young guy from [neighborhood with a large migrant population] who wanted to promote vaccination in [his community]. He grew up there, of course. He’d made these really fun YouTube clips about this and that related to integration, explaining all kinds of things. He interviewed [public health professionals] about vaccination, about how it wasn’t dangerous and all that stuff. And then he explained it in his own way and had a friend asking him questions. I think those kind of things reach so much further than us putting up posters, even if they’re translated. Because it’s coming from one of them. [from interview 8]

Notably, some participants highlighted local sports teams as important partners in community health outreach, recognizing that sports tend to unite people across racial and ethnic barriers and that athletes and coaches are often viewed as authorities on issues related to health and well-being.

However, building trust among communities shaped by migration is not merely about identifying and engaging various community stakeholders that can help in spreading public health information in culturally congruent and relevant ways. The participants emphasized that successful trust-building must also involve a continuous dialogue that allows for community voices to be heard. If the early phases of the pandemic prompted novel ways of speaking to the communities, for health authorities to *listen* to the affected groups might actually have become the most crucial community health outreach component as the pandemic developed. Here, a team of community health workers that were recruited within the various groups with a migration background became important partners, described by one participant as “our ear to the ground”:

So often, we just pour out information from above. But what I appreciated during the pandemic was the dialogue. We met with the community health workers once a week and had a conversation: What do people ask about, what are they worried about? How can we address it? Could we organize a webinar in Somali where people can ask questions? […] I know nothing about the concerns of the Somali group in [neighborhood with a large migrant population]. So they became an important pulse check on people’s worries. [from interview 4]

Several participants expressed that it would have been highly useful to develop and systematize this aspect of working with community stakeholders even further.

#### Addressing real-life concerns in the community

Building on the engagement of community stakeholders, the participants recognized the importance of addressing the very issues that members of the targeted communities saw as most pressing. These issues were typically what might be described as structural barriers related to poor housing, inadequate public transport, insecure employment, and so on. Yet another structural concern was digital exclusion among elderly community members as well as among undocumented migrants, in a time when Swedish healthcare largely relies on online tools for making appointments and receiving feedback on test results. Some participants described this as an awakening of sorts:

It wasn’t about us telling people to act in a certain way. It was about us understanding that it simply wasn’t possible for them to follow our instructions. […] What do you mean “stay at home” when you don’t have a job that allows you to stay home? Most people work in the service sector and they had to go to work, and got infected that way. […] And I think that’s important, because many at our agency couldn’t really understand why people weren’t behaving as expected. [from interview 8]

An increasing focus on structural inequalities sometimes involved interventions that the participants saw as unconventional from a public health perspective, such as reaching out to and collaborating with local public transport authorities. For example, when transport authorities proposed reducing services due to fewer travelers, public health staff urged them to maintain normal service levels so that people could spread out. Notably, although the participants were recruited based on their involvement in designing, organizing, and delivering public health crisis communication, it was obvious that their tasks became much broader during the COVID-19 pandemic as a result of the feedback they received through community dialogue, which prompted them to actively intervene in various ways to try to mitigate the effects of structural inequalities. For many, this was a new experience:

What I’ve thought about afterward is that it was actually less about communication than we ourselves believed. We invest a great deal in communicating various things. But this was really more about considering what we could do beyond that to make it easier for people once they’d received the information—to actually be able to follow what we were telling them to do. [from interview 8]

#### Counteracting stigma

As COVID-19 vaccines became widely available, a main concern was to address and counteract various types of stigma related to vaccination. For example, in order to promote vaccination among those who were undecided, frontline staff described how they made sure to be explicit about the fact that they themselves had taken the vaccine and that it was safe. Sometimes, help in reducing fear would come from unexpected sources:

I went to the police in [neighborhood with a large migrant community]. I know some people there. I asked them to come to the vaccination [spot] if they hadn’t been there already: “Come in your uniforms and get vaccinated for people to see, preferably on a Friday.” And they did. They were three officers. They even took the squad car, a van, and parked in the center. It was a Friday at noon, when people were coming from the mosque. And they said: “Look, the police are getting the vaccine. Let’s get vaccinated too, it can’t be dangerous, the vaccine is safe, we can do it.” I remember that day. A total of 185 people got vaccinated, and they even ran out of vaccine. The following day, there was a long line of people. [from interview 6]

Even so, some participants felt that there was an overemphasis in communicating that everybody ought to get vaccinated, which might have contributed to stigma among those who were worried about potential side effects or undecided for other reasons. It was suggested that an alternative and more viable approach could have been to empathetically acknowledge the various concerns that people might have had and promote informed choice, rather than implicitly shaming those who were reluctant.

### A moving target

The participants stressed that the many uncertainties around the nature of the COVID-19 virus and the optimal pandemic containment strategies—especially during the early phases of the pandemic, but also later on as vaccines started to become available—led to a situation where they felt like they were “chasing a moving target”, “solving a puzzle where the pieces kept moving”, or “building the ship as we sailed it”. This scenario created a continuous need for improvisation in terms of public health communication tactics. Naturally, the ever-changing nature of the pandemic was challenging for most people to handle; even so, the participants highlighted the trust issues among some populations with a migration background discussed above as a clearly complicating factor. For example, inconsistent government messaging during the crisis reminded some migrants of the unreliable and arbitrary information from the authoritarian regimes they had once fled, creating mistrust and emotional distress. The following two subthemes—a need for an overarching strategy and the fostering of a creative atmosphere—explains this further.

#### Need for an overarching strategy

Several participants expressed how rigid organizational structures within the region became a barrier that obstructed community health outreach during the COVID-19 pandemic. This rigidity became particularly notable in relation to pandemic containment as a moving target:

The allocation of responsibility [in public governance] is fairly strict. You do this and we’re responsible for that, and it’s rather well-defined—and whenever something isn’t clear, there are structures in place that tells you who makes that decision. […] Here, it wasn’t really clear who was in charge. Somebody was making plans and procuring buses [for mobile vaccination centers] and other things. And then someone else would say: “That doesn’t work because were doing the same over here [at our department].” Well, who’s running things? [from interview 7]

Here, the participants described how their role as communicators might have given them unique opportunities to identify these internal structural barriers:

The region is very much a silo organization, and that’s something that the communication office often notices: “Hey, why aren’t you two working together, you’re dealing with the same things—hello?” And tries to bridge and connect different things. It’s really an informal [task]. And it’s often discovered when you work in communications. [from interview 4]

In addition to this lack of vertical communication within the organization, several participants experienced a horizontal disconnect between “top-level” public health strategists and the staff “on the grounds”:

I think the problem is with us at [the management level], that we work from a scientific helicopter perspective. And then when you really need to turn it into action, there’s nothing in between. The evidence base and the real-life practice—everything in between is missing. [from interview 4]

For example, one participant mentioned how the public health authorities typically discuss community-level health priorities in terms of a so-called Care Need Index that translates measures of socioeconomic vulnerability into health risk estimates. During the pandemic, however, these usual priorities were sometimes sidelined due to the overall uncertain situation, and the strategies for transforming care needs into community health outreach were not always clear.

#### A creative atmosphere

While acknowledging these difficulties, the participants also highlighted that the need for continuous improvisation fostered a creative atmosphere that was helpful in developing novel outreach strategies and overcoming pessimism. Gradually, successful outreach efforts proved that reaching marginalized or hard-to-reach populations is actually achievable, countering the common defeatist attitude that “it’s impossible” to effectively engage these communities. Not least, when participants were able to challenge and overcome the rigid organizational structures described above, this was experienced as somewhat of a transformative learning opportunity:

Cooperation is kind of a worn-out expression. Sometimes, it just amounts to, well, we have a meeting and you tell me what you’re doing, I tell you what I’m doing, and then we feel as if we have cooperated. But here it was really about doing things together. [from interview 3]

The establishment of a creative, flexible, and hands-on cooperative approach is mentioned as a vital component that ought to be maintained in the post-pandemic context as a benchmark for public health communication and outreach. This is further discussed in the next section.

### Institutionalizing the gains: networks beyond the pandemic

As touched upon above, the participants emphasized how their work in designing, organizing, and delivering public health crisis communication during the COVID-19 pandemic involved cooperative efforts at multiple levels. These efforts included internal cooperation between different regional departments (e.g., public health authorities and public transport authorities) as well as establishing and maintaining networks involving various external agents, such as community centers, religious institutions, property owner companies and housing providers, community business owner associations, street-level youth outreach workers, local sports teams, libraries, news agencies, and other non-governmental organizations. One participant described this as “the birth of a number of important relationships” and it was felt that these semi-formal structures should be maintained in a post-pandemic context, not least as an element of crisis preparedness:

[K]eeping in touch with the local communities, with the neighborhoods or local organizations so that it’s kept in motion, and if something happens you can scale it up instead of starting over again from zero. [from interview 2]

Others underscored how these novel community-level networks focusing on dialogue and cooperation can be highly valuable in working with regular, non-crisis tasks as well. Not least, the insights into structural inequalities affecting communities shaped by migration that have been gained during the COVID-19 pandemic ought to be “kept alive” and allowed to inform routine public health work:

We’ve come pretty far compared to the start of the pandemic. For example, we used to talk about [neighborhood with a large migrant population] as a blank spot on the map. Now there’s a greater understanding, and we also have to understand that we don’t fully understand. That’s the most important thing. You have to recognize the need for learning and for a broader dialogue. [from interview 4]

Notably, it was emphasized that community dialogue should not be seen as something “extra”, but that the approach should be incorporated into the regular regional structures as a standard component. Nonetheless, several participants also expressed how the newly gained insights were quickly being lost as things “returned to normal”. In the words of one participant, “we’ve maintained a visible [community] presence during the entire pandemic, and now we’re back to ignoring it again”.

## Discussion

The findings of this qualitative study, based on interviews with 21 individuals involved in designing, organizing, and delivering public health crisis communication in Stockholm, Sweden during the COVID-19 pandemic, highlight the necessity of moving beyond one-way public health messaging toward an emphasis on promoting dialogue, addressing structural barriers, and establishing a lasting presence and engagement in community outreach. A distinct temporal evolvement was described in the participant narratives. During the early phases of the pandemic, public health authorities in the Stockholm region clearly prioritized linguistic translation of generalized advice for pandemic containment in targeting culturally and linguistically diverse vulnerable populations. While this is certainly a vital component of health crisis communication, it soon became evident that complementary strategies would be necessary; these involved a stronger focus on trust-building activities, identifying and addressing the real-life issues seen as most pressing by the affected communities, and counteracting stigma of various kinds.

This is not necessarily a novel insight in the field of public health communication and outreach. Indeed, in the late 1980s, community development scholar John McKnight posed the following question: “Could it be that for those in greatest need, their health does not depend upon receiving messages? Could it be that their health depends upon controlling the microphone?” [24] It is widely recognized that transferring and building knowledge is not merely equivalent to providing information, but rather determined by the capacity to offer tools and arenas for people to make sense of the information as it relates to their everyday lived experiences [25]. This process of helping people find meaning, manageability, and comprehensibility in information directly reflects Aaron Antonovsky’s influential concept of *sense of coherence* [26]. Even so, there is still an unfortunate tendency in public health communication to frame health risk behaviors as individual and moral choices, which might resulting in victim blaming and stigmatization [27, 28]. While community development advocates point to the need for addressing social determinants of health, governments still typically favor policy actions that aim to change individual-level behaviors through social marketing [29]. This is in spite of the fact that people generally do not engage in health risk behaviors due to a lack of knowledge, but because of life constraints that make them unable or unwilling to act differently [30]. Only in recent years—and perhaps even more so in our post-pandemic society—have previously accepted one-way models of health communication been widely challenged and the importance of identifying blind spots in mainstream public health campaigns increasingly highlighted [31, 32]. Numerous examples of innovative approaches to community engagement during the COVID-19 pandemic have been reported worldwide [33]; however, this focus on social innovation in times of crisis also demonstrates the ad-hoc nature of many of these activities, underscoring the need for partnership-building with vulnerable communities ahead of time.

A further implication of our findings is the need to also reconceptualize trust as a two-way process between authorities and communities. While much attention in public health communication has been devoted to the challenge of cultivating community trust in governance institutions, comparatively less emphasis has been placed on how governance bodies themselves demonstrate trust in local actors. This may take the form of devolving decision-making power, establishing participatory mechanisms, or providing resources that allow communities to lead initiatives on their own terms. By recognizing communities not only as recipients of guidance but as legitimate partners in governance, public health authorities may strengthen both legitimacy and resilience.

In this context, the principles of *participatory action research* (PAR) offer a useful lens for understanding inclusive public health approaches. Rather than positioning communities as passive recipients of information, PAR emphasizes collaboration, mutual learning, and shared ownership of both problems and solutions [34, 35]. A PAR perspective highlights the need to move beyond dissemination toward co-creation, where community members actively shape communication strategies and public health responses. At the same time, sustaining meaningful participation requires long-term commitment and institutional flexibility to avoid tokenism or symbolic involvement [36]. A PAR framework therefore underscores both the opportunities and challenges of working in genuine partnership with marginalized groups. In our data, this tension was visible in the temporal evolvement: initial outreach efforts positioned community members largely as recipients of translated information, whereas over time, community stakeholders—such as trusted local figures and organizations—became active partners in shaping how outreach was conducted, for instance by identifying which structural barriers needed addressing or which messages required reframing to counteract stigma. This shift toward co-creation exemplifies the kind of collaborative, mutual-learning process PAR describes, while also illustrating PAR’s caution around tokenism: several participants noted that such partnerships depended on sustained relationships built over time, rather than one-off consultations.

Our findings also illuminate an important governance dimension: the relationship between top-level strategists and frontline staff. Several participants described a lack of reciprocal channels for communication within the public health system, which resonates with political scientist Michael Lipsky’s scholarship on *street-level bureaucracy* [37] emphasizing the discretionary power of frontline actors in shaping how policies are perceived and enacted. In practice, trust in governance is weakened not only when community members feel unheard, but also when staff themselves perceive a disconnect between organizational directives and the realities of their daily work. Institutional trust therefore depends on governance models that enable continuous feedback loops, ensuring that frontline insights are not only acknowledged but integrated into strategic decision-making. This dynamic was evident in our data in the distinction between senior-level strategists, who designed overarching outreach strategies, and mid-level and frontline staff, who exercised considerable discretion in translating these strategies into concrete action—for example, deciding in the moment how to adapt communication to a specific community’s needs or advocating for structural accommodations, such as maintaining public transit frequency, that fell outside their formal role. Consistent with Lipsky’s framework, this degree of discretion functioned as both a strength, enabling responsiveness, and a source of strain, when frontline staff felt that their on-the-ground insights were not adequately channeled back into strategic decision-making.

Challenges related to siloing within organizations and a need for greater coordination of public health outreach activities were hardly unique for the Stockholm region during the pandemic [38, 39]. In our study, several participants noted that while this experience could be deeply frustrating, it also served as a catalyst for creativity—a tension that has not gone unnoticed in other accounts [40]. Indeed, several insights into creativity emerging from the pandemic have been proposed, including the notion of creativity and standardization as complements rather than antagonists [41]. Nonetheless, our participant narratives suggest that creativity often became essential in the face of missing or inadequate structures. Thus, what is described as a creative atmosphere can also be framed in terms of *ad hocery*, a phenomenon which is known to be associated with underperformance in crisis management [42].

The perceived urgency for creativity and innovation during the COVID-19 pandemic may also be understood as a reflection of the absence of preexisting policy measures addressing the needs of vulnerable populations. This is in spite of well-established empirical knowledge about the impact of minoritization on health, access to healthcare services, and crisis psychology. Public health scholars Michelle Amri and Don Drummond suggest that, simply put, “attention should not be solely directed to flash fires, but instead, to the bushes that were burning before the crisis and will burn long after” [43]. Through an optimistic lens, experiences from the COVID-19 pandemic clearly demonstrate a capacity within public health authorities to act boldly in the face of a health crisis. Significant attention must now be given to fostering and maintaining ongoing recognition of the various social determinants of health as well as the importance of community partnerships in non-crisis times, in order to strengthen preparedness for future health crises or other civic society crisis. Importantly, *punctuated equilibrium theory*—originally developed by political scientists Frank Baumgartner and Bryan Jones—predicts how constructive public policy change can occur in response to a major event or crisis after prolonged periods of stability or stagnation [43, 44]. However, such windows of opportunity for reform are typically brief and may close quickly if momentum is not sustained, returning policy systems to their previous equilibrium state.

Indeed, experience demonstrates that authorities often fail to learn from past major incidents, despite their best intentions [45]—an ambivalence clearly expressed in our theme “Institutionalizing the gains: networks beyond the pandemic”. Participants described the pandemic as having catalyzed valuable new cross-sectoral relationships and ways of working, yet several also expressed concern that these gains were fragile and at risk of dissipating as institutional attention returned to pre-pandemic routines, precisely the kind of reversion to a prior equilibrium the theory anticipates. The question of whether governance systems are able to learn from crises has direct implications for public trust. As noted by some of our participants, institutional memory is often short-lived and vulnerable to staff turnover, with many of the same challenges recurring despite official inquiries or stated commitments to reform. When lessons identified do not translate into lessons learned, public perceptions of governance are at risk of being undermined. Conversely, transparent acknowledgement of missteps, coupled with mechanisms of accountability and reform, can contribute to repairing and even strengthening trust. In this respect, post-crisis periods should be seen not only as moments for policy innovation, as suggested by punctuated equilibrium theory, but also as opportunities for institutions to demonstrate their credibility through sustained commitment to organizational learning.

The patterns reported here resonate with findings from studies conducted in other national contexts. In Norway, qualitative research similarly found that translated information, while available, did not always reach migrant populations effectively, pointing to a gap between availability and actual accessibility [6]—paralleling our own theme on the promise and limits of language-based outreach. The same study found that information delivered through everyday institutions such as schools and workplaces was often better received than communication explicitly targeted at migrant identity, resonating with the broader emphasis in public health communication scholarship on anchoring information in people’s everyday lived experience rather than messaging aimed at migrant status alone. Relatedly, a separate Norwegian study on a health ambassador model—in which trusted members of migrant communities were engaged to disseminate health information to their peers—found that while ambassadors generally experienced their role as meaningful, several also reported psychological distress associated with the responsibility of conveying accurate information and countering misinformation, as well as spending more of their free time than expected assisting others in practical ways [46]. This underscores that engaging community members as communication intermediaries, while valuable, may also place unanticipated burdens on those individuals that warrant consideration in future outreach initiatives.

A study of Muslim communities in North West England similarly found that elevated COVID-19 risk among these Black and minority ethnic populations was attributable largely to structural factors, particularly occupational exposure, rather than to gaps in awareness or understanding [47]. This is consistent with our own observation that dialogue-based engagement helped outreach staff identify and understand the structural barriers, rather than mere information gaps, underlying persistent disparities in COVID-19 outcomes among migrant and minoritized populations in Sweden. Comparable dynamics have been documented beyond Europe: in a qualitative study of the Ezidi refugee community in rural Australia, government-issued COVID-19 information was widely perceived as poorly tailored to community needs, whereas trusted local individuals and service providers played a more decisive role in shaping message uptake, leading the authors to recommend that government health services partner directly with such trusted intermediaries [48]. Taken together, these examples—a small selection from a growing international literature—point to a shared policy imperative: complementing translation-based outreach with sustained dialogue that can surface, and ultimately help address, the structural barriers underlying persistent health disparities.

### Rigor and trustworthiness

This study contributes to a small but expanding body of literature that underscores the importance of dialogue and deeper engagement with communities shaped by migration, drawing on lessons from the COVID-19 pandemic. A strength of our study is the broad perspective on public health outreach, involving participants engaged in designing, organizing, and delivering health crisis communication activities at various levels within the regional public health authorities and healthcare services during the COVID-19 pandemic. This heterogeneity allows for the triangulation of multiple voices; however, it could also possibly be regarded as a limitation in that the viewpoints of any specific subgroup of participants are not explored in full depth.

Furthermore, participants were recruited in part through existing collaborative relationships with the Transcultural Center, which may have introduced a degree of selection bias: individuals who had already engaged in collaborative outreach work may be more inclined toward community engagement and cross-sectoral communication than staff without such prior involvement. As a result, the experiences and perspectives captured in this study may not fully represent those of staff who were less engaged in, or more skeptical of, outreach-oriented approaches to crisis communication. However, we sought to invite nearly all individuals who had been involved in the relevant outreach activities, rather than a selective subset, which may partially mitigate this concern.

Another potential limitation of this study concerns the combined use of individual and focus group interviews. While focus groups are typically employed to elicit interactional dynamics among participants—such as disagreement, negotiation, or consensus-building—our use of this method was driven by practical considerations related to participant availability rather than an aim to capture group dynamics. As a result, our analysis focused on content shared across both individual and group data rather than on interactional processes specific to the focus group format.

In the interview procedure, measures were taken to reduce the risk of informant bias. In order to mitigate any potential tendencies among participants to exaggerate the success of the regional public health outreach efforts during the pandemic, the interviews explicitly focused on areas of potential improvement for future health crises. It was also clearly emphasized that the individual professional performance of the participants during the pandemic was not under evaluation in the study. Moreover, the participants were not in a dependent position vis-à-vis the interviewers. It is our general impression that the participants did not express any concern in voicing potentially critical opinions and that the interview data included a wide range of insights on positive and negative aspects of the regional pandemic containment strategies. Nevertheless, the risk that some participants did not feel comfortable sharing their viewpoints cannot be entirely ruled out.

Regarding the transferability of our findings, we note that the study was conducted in a specific regional context—i.e., Stockholm county—characterized by particular administrative structures, a specific composition of migrant and ethnically minoritized communities, and a distinct pandemic response trajectory shaped by Swedish public health policy. As such, the specific outreach strategies and organizational barriers described here may not directly generalize to other regional, national, or health system contexts. However, the broader analytic themes identified—namely, the limitations of translation-only approaches, the value of trust-building and community dialogue, the importance of addressing structural barriers, and the risk of losing institutional learning once a crisis subsides—reflect processes that are plausibly relevant across a range of public health crisis contexts beyond COVID-19 and beyond Sweden. We therefore suggest that while the specific empirical findings are context-bound, the underlying analytic insights may hold transferable value for public health authorities operating in other settings facing similar structural and communicative challenges.

## Conclusion

The findings reported here underscore the critical need for public health authorities in Region Stockholm, and likely elsewhere, to fundamentally shift from traditional one-way messaging approaches toward genuine community partnership and dialogue-based engagement. Effective crisis communication requires not only linguistic translation but also trust-building, addressing structural barriers, and maintaining a sustained community presence. The evolution over time described by participants—from initial focus on translated materials to recognition of the need for deeper community engagement—reflects broader challenges in public health communication that extend well beyond pandemic responses. This study highlights how the tendency to treat communities as passive recipients rather than active partners can undermine both the effectiveness of public health interventions and community trust in governance.

Looking forward, several key implications emerge for strengthening public health preparedness and community resilience. First, our findings emphasize the urgency of building long-term sustainable community partnerships and addressing social determinants of health during non-crisis periods, rather than relying on ad-hoc solutions during emergencies. As suggested by punctuated equilibrium theory, while crises create windows for policy innovation, these opportunities are fleeting and require sustained institutional commitment. Second, governance systems must develop robust mechanisms for organizational learning that translate crisis experiences into lasting structural changes, including improved coordination across agencies, enhanced feedback loops between frontline workers and decision-makers, and institutionalized community participation. Without such sustained efforts, public health authorities risk repeating past mistakes and eroding public trust—a particularly concerning scenario given the likelihood of future health crises and the growing importance of community cooperation in addressing complex health challenges.

## Supplementary Information

Below is the link to the electronic supplementary material.

Supplementary Material 1

## Data Availability

The dataset used during the current study is available from the corresponding author in fully anonymized form on reasonable request.

## Abbreviations

COVID-19: Coronavirus disease 2019
PAR: Participatory action research
