# Supplementary material for: “We built the ship as we sailed it”: exploring public health outreach targeting communities shaped by migration in Stockholm, Sweden during the COVID-19 pandemic
Source: BMC Health Serv Res. 2026 Aug 18;26:1154. doi: 10.1186/s12913-026-15396-z (PMC13488172; doi:10.1186/s12913-026-15396-z)
Supplement: Supplementary file 1 [file 12913_2026_15396_MOESM1_ESM.docx]

**Supplementary Material: Interview Guide**

The questions in this semi-structured interview guide are intended to help the interviewer maintain a topical focus during the interview. They do not need to be asked verbatim or in a fixed order. Questions that are not relevant for a particular participant may be skipped, and the interviewer may also introduce supplementary questions not listed here, depending on the participant’s narrative.

- What has your role been in Region Stockholm’s efforts to disseminate information about COVID-19 and vaccination to people whose first language is not Swedish?
- How have you worked with communication in this context?
- How has your work changed over time?
- What positive experiences have you had in reaching the target group of people whose first language is not Swedish?
- What have been the main challenges in reaching the target group?
- How have you worked to overcome any challenges of reaching and communicating with the target group?
- Which partners or collaborators have been important in this work?
- What organizational factors have been important for enabling effective communication?
- What organizational factors have made the communication work more difficult?
- What experiences have you had with media contacts regarding Region Stockholm’s communication work about COVID-19 and vaccination aimed at people whose first language is not Swedish?
- What lessons would you like to highlight for future communication efforts aimed at people whose first language is not Swedish in the event of a new health crisis?
- Looking back at Region Stockholm’s communication efforts aimed at people whose first language is not Swedish, what do you think should have been done differently—or should be done differently next time if a new crisis arises?
